# The Effects of Abutment Finish Lines on the Penetration Characteristics of Elastomers into the Simulated Gingival Sulcus

**DOI:** 10.1055/s-0042-1759697

**Published:** 2023-01-04

**Authors:** Atiwat Jaroenpiboon, Pongsakorn Apinsathanon, Pheeradej Na Nan, Napapa Aimjirakul

**Affiliations:** 1 Department of Conservative Dentistry and Prosthodontics, Faculty of Dentistry, Srinakharinwirot University, Bangkok, Thailand; 2 Private Practice, PSK Dental Center, Bangkok, Thailand; 3 National Cyber Security Agency, Bangkok, Thailand

**Keywords:** dental, abutment, finish line, impression materials, penetration ability

## Abstract

**Objective** The aim of this study was to determine the effect of finish lines on the penetration ability of polyether and polyvinyl siloxane impression material into the simulated gingival sulcus.

**Materials and Methods** Three types of finish line (chamfer, deep chamfer, and radial shoulder) were impressed with two types of elastomeric impression material (polyether and polyvinyl siloxane) using a two-step impression technique. Ten samples of each finish line were prepared and then separated into two groups of impression material: polyether and polyvinyl siloxane. The model of the simulated gingival sulcus had a width of 0.1 mm and a depth of 3.5 mm with a subgingival finish line of 0.5 mm. The effect of the finish lines on the penetration ability of these impression materials was analyzed using a two-way analysis of variance (ANOVA) and Tukey's multiple comparison tests at a statistically significant level of 0.05.

**Results** A two-way ANOVA revealed a significant difference among finish lines, impression materials, and their interaction. The deep chamfer and radial shoulder finish lines displayed significantly higher penetration ability than the chamfer finish line. Moreover, polyether revealed significantly higher penetration ability than polyvinyl siloxane.

**Conclusion** The finish lines affected the penetration ability of the impression materials. Therefore, the simulated gingival sulcus model demonstrates that it is an effective way of examining impression materials' penetration abilities.

## Introduction

Fixed dental prostheses have become a very popular choice of treatment for either replacing or restoring teeth. Young adult patients choose fixed dental prostheses more frequently than removable dentures. 1 Fixed dental prostheses require good quality dental imprints with accurate details of tooth abutments, especially regarding the finish line positions. 2

In fabricating a fixed dental prosthesis, dentists must prepare sufficient space around the abutment tooth for the restorative material. 3 4 The finish line characteristics should be: (1) easy to prepare without overextending and unsupported enamel; (2) easy to locate during the clinical and laboratory process; (3) able to provide adequate thickness for the restorative material; and (4) able to conserve the tooth structure. 5 In the majority of cases, dentists use three types of finish lines for crown and bridge restorations: (1) a chamfer finish line for a full metal crown; (2) a deep chamfer finish line; or (3) a radial shoulder finish line for a porcelain fused to metal crown and for all ceramic crowns. 5 6 7 Partially because of biological considerations and maintenance, supragingival finish lines are recommended. 5 However, in certain cases, subgingival finish lines may be used for a lesion that extend the subgingival area or for esthetic reasons. 5 8 9

Elastomeric impression materials—especially polyether and polyvinyl siloxane—are widely used during the final impression step. 10 When using the gingival retraction technique during this final impression step, a sulcular width of more than 0.2 mm is recommended. 11 When gingival retraction cords are removed, the sulcular width rapidly relapses. 12 Narrowing of the gingival sulcus width inhibits the penetration ability of impression material into the sulcus, therefore making it difficult to define the location of finish lines on the tooth abutment. 13

Impression materials can help achieve accurate duplication of tooth abutments. Three properties that affect the penetration ability of impression materials into the gingival sulcus are thixotropy, rheology, and wettability. 14 15 16 Thixotropy is a shear-thinning property that is time dependent. When shear stressed, certain gels or fluids that are viscous under static conditions will flow over time. 5 The rheology property is the science of flow and deformation of matter, with respect to both solids and liquids. It is relevant to the relationships between shear stress, shear strain, and time. 17 The wettability property is the measurement of a liquid's ability to interact with other fluids and/or a solid surface. Wettability measures the level of wetting when solid and liquid phases interact with each other via the contact angle. Typically, a 90-degree contact angle is considered as a threshold value. Wettability is lower when the contact angle of water is above 90 degrees, referred to as “hydrophobic,” and higher when the contact angle is below 90 degrees, which is referred to as “hydrophilic.” 18

The aim of the present study was to use the simulated gingival sulcus model to assess the penetration ability of two elastomeric impression materials, polyether and polyvinyl siloxane, on three types of finish lines, namely, chamfer, deep chamfer, and radial shoulder finish lines.

## Materials and Methods

This experimental study used the simulated gingival sulcus model to assess the penetration ability of two elastomeric impression materials, polyether and polyvinyl siloxane in two consistencies: heavy body and light body as shown in Table 1 , on three types of finish lines, namely, chamfer, deep chamfer, and radial shoulder finish lines. This experimental study was performed following ISO 4823:2021(dentistry–elastomeric impression and bite registration materials). 19 A total of 30 impressions were made from the simulated sulcus models, 5 impressions using each impression material for each of the three finishing line groups.

**Table 1:** Elastomeric impression materials

Type of impression material	Trade name	Consistency	Lot. number
Polyether	Impregum 3M ESPE, Seefeld, Germany	Heavy body	5023235
		Light body	7478882
Polyvinyl siloxane	Imprint 4 3M ESPE, Seefeld, Germany	Heavy body	7425293
		Light body	7369858

### Model Preparation

**Fig. 1:**
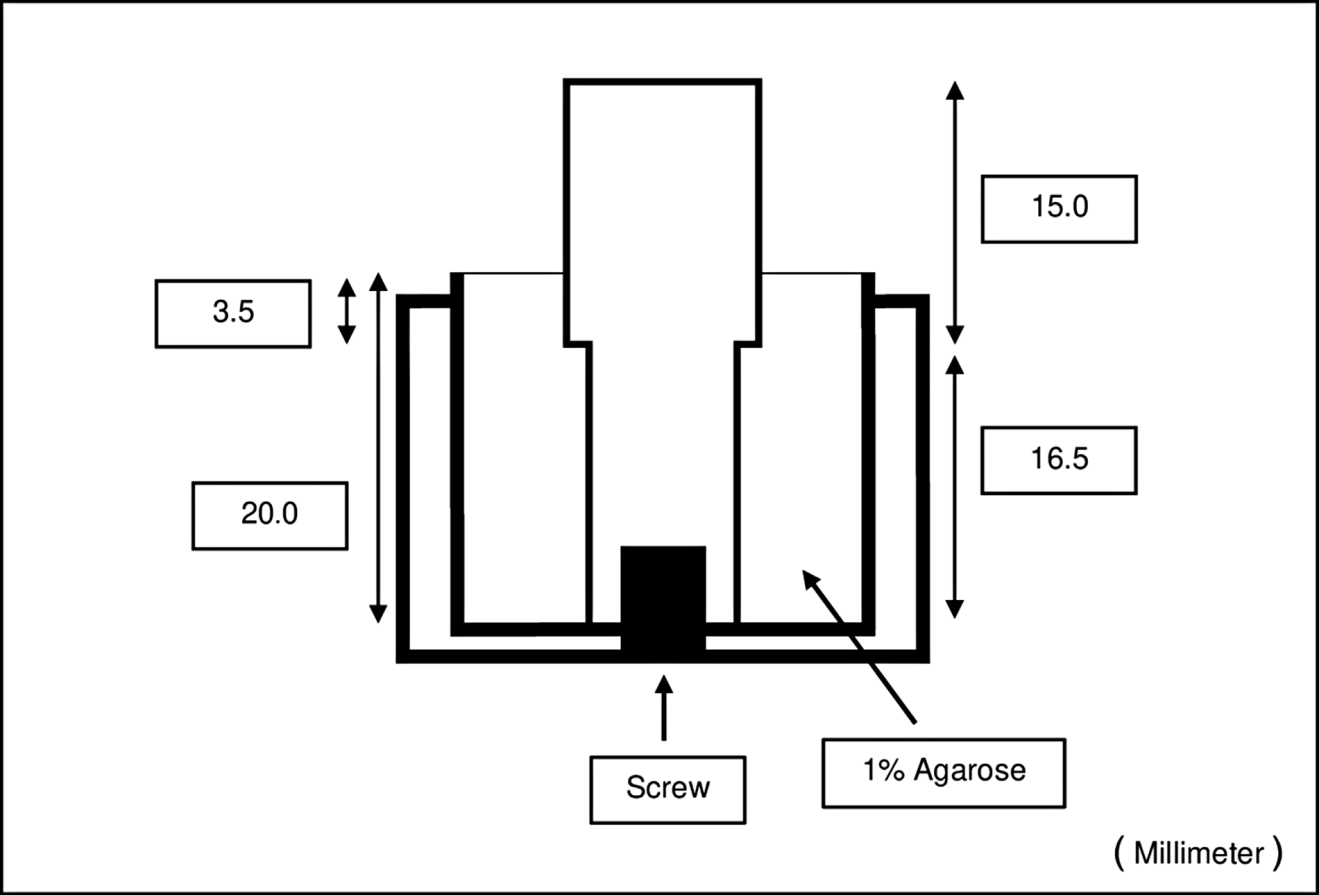
Model preparation of simulated gingival sulcus with the cylindrical stainless steel model.

The cylindrical stainless steel model (upper part size: diameter 8.650 mm, height 15.000 mm; lower part size: diameter 8.450 mm, height 16.500 mm) was fabricated using a computer numerical controlled milling machine. The model was fixed into the plastic block with a screw ( Fig. 1 ). The simulated gingival sulcus was made by using 1% agarose gel (LE agarose, SBIO, Smart science co., Ltd, Thailand, Lot. D00199). The gel was poured into the plastic block and incubated for 30 minutes at 35 ± 1°C with 100% humidity. Next, the cylindrical stainless steel model was removed and replaced by the experimental finish line stainless steel model (upper part size: diameter 8.450 mm, height 8.015 mm with a taper of 6 degrees) with three types of finish line: (1) chamfer, width 0.5 mm, (2) deep chamfer, width 1.0 mm, and (3) radial shoulder, width 1.0 mm (lower part size: diameter 8.450 mm, height 19.500 mm) in a plastic block with a screw.

**Fig. 2:**
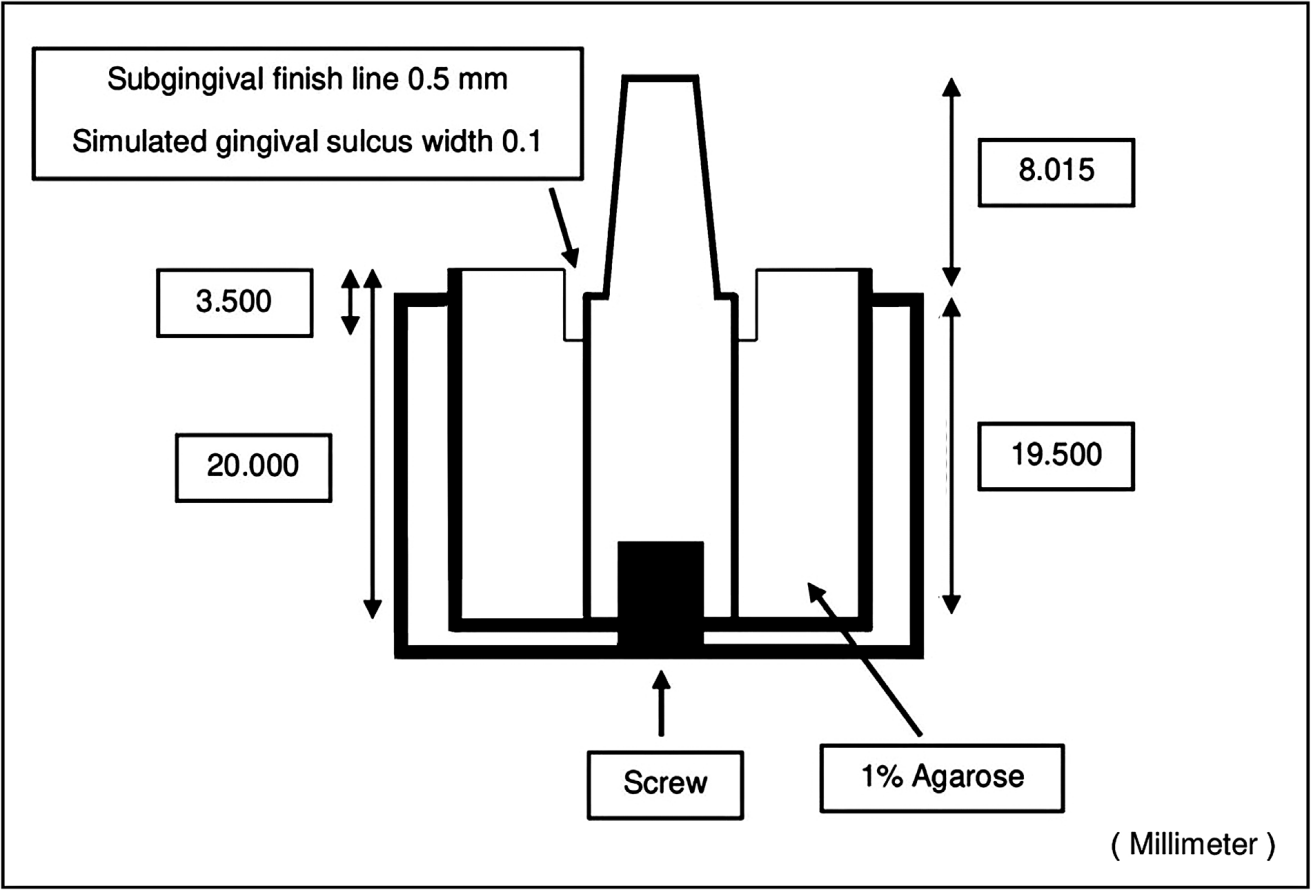
Simulated gingival sulcus with the radial shoulder finish line stainless steel model.

The simulated gingival sulcus model was 0.1 mm in width, 3.5 mm in depth, and the subgingival finish line was 0.5 mm ( Fig. 2 ). The model of simulated gingival sulcus was used immediately.

### Penetration Ability Test

**Fig. 3:**
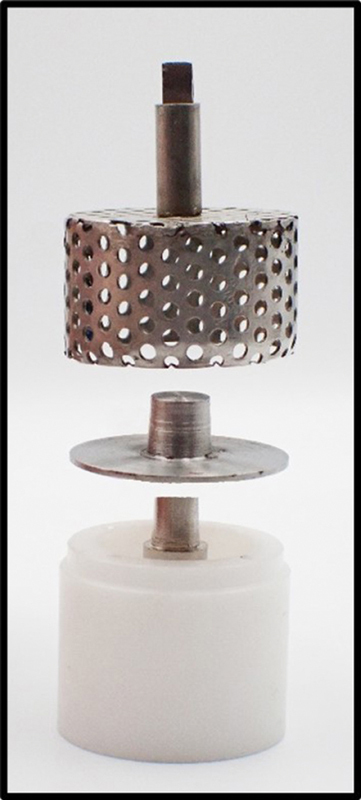
Impression tray, stainless steel cap, and simulated gingival sulcus model.

The three types of finish lines (chamfer, deep chamfer, and radial shoulder) were impressed with two types of impression material: polyether and polyvinyl siloxane, using a two-step impression technique ( Fig. 3 ). Ten samples of each finish line were used and these were separated into two groups using simple random sampling for each type of impression material. The first impression was impressed with the heavy body and the second impression was impressed with the light body under the universal testing machine (EZ test; Shimadzu Corporation, Kyoto, Japan) at a speed of 500 mm/min. The impression trays were removed and stored for 24 hours before measuring the penetration of the impression materials.

### Data Collection

**Fig. 4:**
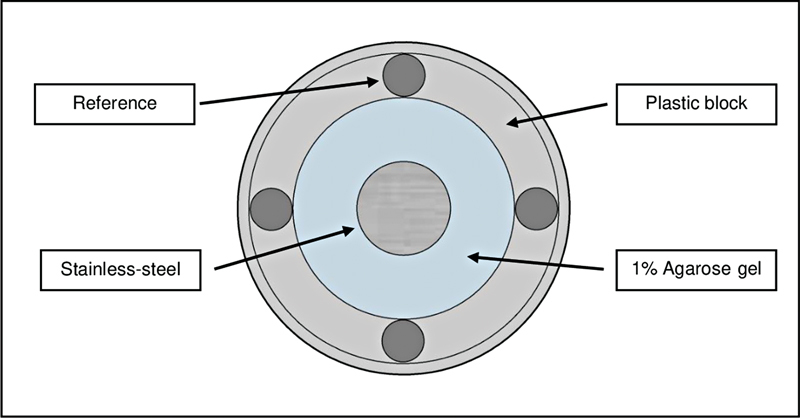
Four reference points on the plastic block.

The penetration ability (creeping of impression material into the simulated gingival sulcus model) was measured in millimeters, using a measuring microscope (MM-11; Nikon, Tokyo, Japan) set at ×10 magnification. The penetration ability of each sample was calculated according to four reference points at the margin of the plastic block ( Fig. 4 ) which was replicated by the impression material.

### Statistical Analysis

The data were analyzed using a two-way analysis of variance (ANOVA) with IBM SPSS statistics 21, at a significance level of 0.05. Mean differences were determined using Tukey's multiple comparison tests.

## Results

**Fig. 5:**
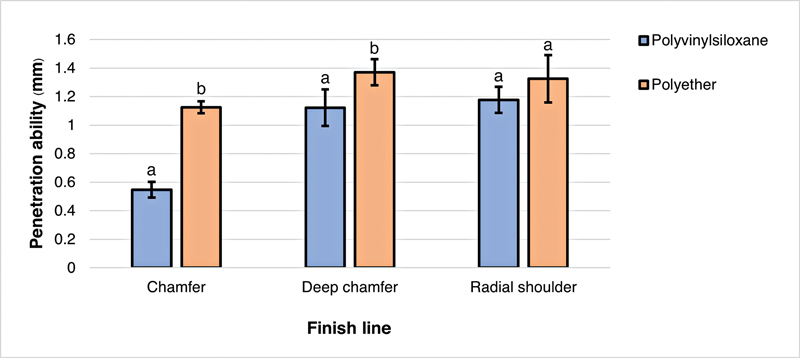
Mean penetration ability of each impression materials. The same lowercase superscript letters indicated no significant differences between the types of impression material in the same finish line.

The effects of finish lines on the penetration ability of impression materials are shown in Table 2 , that is, the mean values and standard deviations of the penetration ability of polyether and polyvinyl siloxane into the stimulated sulcus. The mean values were normally distributed for all groups (Shapiro–Wilk's test, *p* > 0.05). Levene's test for homogeneity of variance was not significant across group ( *p* = 0.054). A two-way ANOVA revealed significant main effects for finish lines and the impression materials; importantly, the interaction between finish lines and impression materials was also statistically significant ( *p* < 0.05). Post hoc test (Tukey's multiple comparison test) revealed that for both impression materials, the deep chamfer and the radial shoulder finish lines displayed significantly higher penetration ability than the chamfer finish line. In addition, polyether produced significantly higher penetration ability than polyvinyl siloxane for both the chamfer and the deep chamfer finish lines; however, there was no significant difference between these impression materials for the radial shoulder finish line ( *p* > 0.05) ( Fig. 5 ). For polyvinyl siloxane, the radial shoulder finish line shows greater penetration ability, followed by the deep chamfer finish line and the chamfer finish line. However, for polyether, the deep chamfer finish line has the greatest penetration ability, followed by the radial shoulder and chamfer finish lines. Considering dental impression material, the results show that polyether has greater overall penetration ability than polyvinyl siloxane.

**Table 2:** Mean penetration ability in millimeters of each impression material (standard deviations in parentheses)

Finish line	Polyvinyl siloxane	Polyether
Chamfer	0.546 (0.055) ^A,a^	1.125 (0.041) ^A,b^
Deep chamfer	1.121 (0.128) ^B,a^	1.370 (0.091) ^B,b^
Radial shoulder	1.176 (0.091) ^B,a^	1.325 (0.166) ^B,a^

Note: Groups with the same uppercase superscript letters indicated no significant differences between the types of finish line in the same impression material at *p* < 0.05. Groups with the same lowercase superscript letters indicated no significant differences between the types of impression material in the same finish line at *p* < 0.05.

## Discussion

In this study, we have shown that the type of abutment finish line and impression material affects the penetration ability of the impression material. The results revealed that deep chamfer and radial shoulder finish lines displayed higher penetration ability than chamfer finish lines. Regarding the impression material, polyether produced higher penetration characteristics than polyvinyl siloxane.

Typically, chamfer finish lines are used for metal crowns, whereas deep chamfer and radial shoulder finish lines are used for porcelain fused to metal crowns and for ceramic crowns. The reason is that the width of finish line has to be sufficient for the thickness of the restorative material. 5 6 7 Nowadays, a novel zirconia crown is acceptable with a chamfer finish line preparation. 20 In terms of marginal fit, Subasi et al 21 revealed no significant difference between the marginal fit of the chamfer finish line and the rounded shoulder finish line when restored with IPS e.max Press or Zirkonzahn. By contrast, Faruqi et al 22 showed that heat-pressed lithium disilicate crowns revealed a better marginal fit than both layered zirconia and monolithic zirconia crowns, and the chamfer finish lines revealed a better marginal fit than the shoulder finish lines. According to clinical studies, the type of finish line has no effect on the clinical outcome of fixed dental prostheses. However, dentists must prepare enough space on the abutment tooth for the restorative materials. 3 4

There are three techniques for the final impression step: single viscosity, dual viscosity, and two-step impression technique. This study used the two-step impression technique which has low shrinkage and completely polymerized high viscosity impression material that is pressed over a low viscosity impression material into the sulcus. 23 24 In addition, this technique shows a higher penetration of impression material into the sulcus compared with other techniques. 25

Ideally, the impression materials should be nontoxic with high accuracy and no gas release. They also need to be hydrophilic with stability, viscosity, flexibility, and tear resistance. For dentists, it is important that these materials have a long shelf life and are low cost. 24 They also need to be easily disinfected and compatible with model pouring materials. 24 26 27 In addition, the materials should provide dentists with adequate working time to manipulate easily during a treatment procedure. For patients, impression materials should have pleasing flavors, odor, and color. 24

At present, the elastomeric impression materials such as polyether and polyvinyl siloxane are widely used as final impression materials. These impression materials will penetrate into the sulcus and imitate the tooth abutment with the penetration ability consisting of three properties: thixotropy, rheology, and wettability. 14 15 16 Thixotropy is when a liquid or gel acquires shear stress and causes a decrease in viscosity but an increase in its flow ability. 5 A previous study by Martinez et al 14 revealed that both Imprint II pastes (base and catalyst pastes) have yield stresses of approximately 40 Pa in addition to some real thixotropy. By contrast, while both Examix pastes (base and catalyst pastes) produced no yield stress value, the catalyst paste was thixotropic as seen by a decrease in viscosity as shear force was applied over time. Martinez et al 14 concluded that both Imprint II and Examix polyvinyl siloxane have thixotropic properties. From this study, the deep chamfer and radial shoulder finish lines show higher penetration ability than the chamfer finish line because of the thixotropic properties of the impression material. The deep chamfer and radial shoulder finish lines have a wider width of an abutment tooth, which creates greater shear stress and effects on the thixotropic properties of the impression material than the chamfer finish line. Shear stress occurs while injecting the impression material around the abutment tooth and during the seating of the tray. In addition, the thixotropic property will help prevent the overflow of material once it has been injected around the abutment tooth until the impression tray is loaded and seated. 14 28

The rheological property is related to the ability of impression materials to flow. Elastomeric impression material has increased viscosity and elasticity after mixing. Polyvinyl siloxane is the additional reaction of polymer, between divinylpolysiloxane and polymethylhydrosiloxane with a platinum salt catalyst. Polyvinyl siloxane has a static polymerization reaction after mixing base and catalyst pastes according to the amount of catalyst included by the manufacturer. By contrast, polyether is a cationic polymerization reaction of polymer, between polyether molecules and aromatic sulfonate ester initiators. The catalyst will be released in increasing quantities during the cationic polymerization reaction, which makes the polymerization reaction terminate rapidly in the setting time phase that is called the “snap set” property of polyether. 15 18 29 30 The results of the present study shows that polyether has higher penetration ability than polyvinyl siloxane, which is compatible to a previous study by German et al. 15 During the working time phase, polyether has a slow increase in viscosity and elasticity, which allows it to achieve greater penetration into the sulcus than polyvinyl siloxane. Furthermore, polyvinyl siloxane, Imprint 4, contains a monofunctional UCS setting accelerator, which can increase in temperature after mixing. Mccabe and Arikawa 30 examined the rheological properties, the loss tangents and the dynamic viscosities, of elastomeric impression materials. After mixing, the polyvinyl siloxane rapidly becomes elastic, while the polyether retains its plasticity for a certain time. Additionally, an increase in temperature results in a shorter working time and setting time for elastomeric impression materials. The present results are also consistent with those found by Mccabe and Arikawa 30 and German et al. 15 The very high value of flow determined by the shark fin test is explained by the high tan delta of polyether at the working period. In addition, the height of the shark fin for polyether was an order of magnitude greater than that for the other polyvinyl siloxane materials.

The results show that polyether had higher penetration ability than polyvinyl siloxane. Although the properties of polyether are similar to polyvinyl siloxane, polyether is more hydrophilic. 31 32 Consequently, polyether can penetrate in moist conditions via ester functional groups (R-CO-OR) that are slightly positive polar molecules, that is, dipole–dipole force with water molecules. 16 18 31 On the other hand, polyvinyl siloxane is hydrophobic by nature. Hydrophilic polyvinyl siloxane contains a surfactant (e.g., polyalkylene oxide), which allows polyvinyl siloxane to penetrate in moist conditions. 16 18 33 The stimulated gingival model made from reversible hydrocolloid (1% agarose gel) replicates the moist conditions of the oral cavity. 13 15 Menees et al 16 showed that hydrophilic polyvinyl siloxane (Imprint 4) and hybrid impression material (Identium) had the lowest contact angles, while the polyether (Impregum) was intermediate, and the traditional polyvinyl siloxane revealed the highest contact angles when tested with water. For the saliva test, Identium, Impregum, and Imprint 4 were in the group with the lowest contact angle. Nassar et al 33 reported the contact angle of water on set elastomeric impression materials from 0 to 60 seconds was 41.2 to 10.1 degrees for hydrophilic polyvinyl siloxanes (Imprint 4), 83.7 to 40.7 degrees for vinylsiloxanether (EXA'lence), and 71.8 to 40 degrees for polyether (Impregum). However, Takahashi and Finger 34 revealed that there is no difference in the reproduction of surface detail of moisture dentine when impressions were made with hydrophobic or hydrophilic impression materials. In contrast to the present study, Johnson et al 35 reported that polyether demonstrates better reproduction of detail than hydrophilic polyvinyl siloxane in moist conditions. This is the result of the truly hydrophilic of polyether. 16 18 31 35 However, dry conditions were recommended for the best reproduction of detail. 5 35 The results from the present study also show that polyether has a high penetration ability not only due to its wettability but also because of its rheology and thixotropy properties. 34 35

The shark fin test is a penetration test designed and developed by 3M ESPE to demonstrate the flowability of polyether impression materials into narrow spaces. 15 36 37 A new method was designed and introduced with simulated gingival made from reversible hydrocolloid (1% agarose gel) to study the penetration characteristics of impression materials in moist conditions. 13 38 In the previous studies (e.g., Aimjirakul et al 13 ; Apinsathanon et al 38 ; Suwanwalaikorn et al 39 ), the results indicate that polyether can penetrate into the simulated sulcus further than polyvinyl siloxane and other impression materials, regardless of sulcular width, which was similar to the results of the present study. The stainless steel models with a 6-degree taper, 0.5-mm chamfer finish line, 1.0-mm deep chamfer finish line, and a 1.0-mm radial shoulder finish line were the ideal tooth preparation for dental crown restoration, and the agarose was a good representation of gingiva in moist conditions. In addition, sulcular width, position, and type of finish lines can be modified to study the penetration ability of the elastomeric impression material. The model in this study was modified from a patent granted in 2020 by the Department of Intellectual Property in Bangkok, Thailand. 40

The three properties: thixotropy, rheology, and wettability can affect the penetration ability of impression materials. 14 15 16 For clinical applications, a narrow gingival sulcus situation can be achieved by preparing deep chamfer or radial shoulder finish lines, with polyether recommended as the impression material. However, dentists must consider the remaining tooth structure or tooth and tissue undercut before deciding on the treatment options. In addition, clinical studies as well as laboratory studies of other types of finish line, impression material, and gingival sulcus width should be further examined.

## Conclusion

Within the limitations of this study, it can be concluded that the abutment finish lines affect the penetration ability of elastomeric impression materials. Polyether has a higher penetration ability than polyvinyl siloxane regardless of abutment finish lines. In addition, the current simulated gingival sulcus model is effective in evaluating the effect of abutment finish lines on the thixotropic properties of elastomeric impression materials
